# Peer Review of “Telerehabilitation for People With Physical Disabilities and Movement Impairment: A Survey of United Kingdom Practitioners”

**DOI:** 10.2196/35853

**Published:** 2022-01-03

**Authors:** Maria Stella Stein

**Affiliations:** 1 School of Allied and Public Health Professions Faculty of Medicine, Health and Social Care Canterbury Christ Church University Canterbury, Kent United Kingdom

**Keywords:** telerehabilitation, physical disabilities, movement impairment, remote assessments, telehealth, rehabilitation, training, health care practitioners, physiotherapy, occupational therapy

*This is a peer-review report submitted for the paper “Telerehabilitation for People With Physical Disabilities and Movement Impairment: A Survey of United Kingdom Practitioners.”*

## Round 1 Review

### General Comments

The content of this paper [1] is of interest to the journal readership especially post COVID-19 pandemic and the rapid move to online practice in the rehabilitation field. It is reasonable to assume that online rehabilitation interventions are here to stay albeit to a different extent than during the pandemic. The manuscript as it stands reads well; however, the quality can be further improved by considering the following.

### Specific Comments

Please be consistent with terminology, either the authors use “in person” or “face to face” but avoid using both terms to refer to the same method. Preferable to free text and fixed option, consider replacing with open and closed ended questions; it reads more professional.

#### Main Comments

##### Title

Insert the word “interventions” next to Telerehabilitation.

##### Abstract

The Results section could be further summarized. Suggest referring to *challenges* rather than *obstacles*.

##### Introduction

There is a reasonable introduction that could be further supported with actual figures. For example, how common are the physical disabilities being referred to? Include an operational definition of physical disabilities. This would normally include motor impairment, so why does the paper refer to physical disabilities and movement impairment. I think this needs clarification supported by the literature.

It would also strengthen the rationale for the study if slightly more context were provided for key studies cited in this section [2-7].

##### Methods

1. Design and development: the first sentence should read “findings from the scoping review...” The authors refer to “experts,” please indicate which experts these were.

Second paragraph: this sentence does not read well or make sense on its own: “To maximise accuracy and completeness of data, formatting and compulsory items [8] were used in the questionnaire design.” Suggest rewriting or providing a little more explanation.

Third paragraph: re: questionnaire: How long was the estimated time of completion? Could the same respondent complete it a second/multiple times? Were any measures in place to prevent this from happening?

Make it clear that the questionnaire was anonymous but with an option for contact details if the respondent chose to include these.

2. Recruitment and data collection: as a general comment, the selection criteria are not clearly explained. For example, who was classified as a rehabilitation practitioner and therefore could participate in the survey? Were there any measures in place to check that respondents were genuinely professional people (ie, verification of identity)?

Were there any exclusion criteria?

Clarify consent: was this if they returned the completed survey, then it was taken as automatic consent?

3. Data analysis: Delete this sentence: “No statistical correction (such as weighting of items or use of propensity scores) was used; this was not felt to be appropriate as this was not a probabilistic sample.” It is redundant.

##### Results

The authors write “Of the 247 respondents, 207 (84%) reported having used video-based consultations.” The reviewer is wondering why did the other 40 not use video consultations. Was this not an inclusion criterion? Please explain.

Further down, the authors write “In free text responses, reduced travel and improved flexibility were deemed particularly beneficial for those with physical disabilities and fatigue.” Consider referring to open ended questions instead of free text. Additionally, clarify who benefited from reduced travel and improved flexibility—does this refer to professional, client, or both?

The next sentence refers to multidisciplinary working. Please explain which aspects pertain to being multidisciplinary (eg, communication or decision-making).

Figure 2: The title refers to perceived benefits, please clarify for whom? Is this written from a professional perspective, as only professionals completed this survey? It is important to make this distinction.

Consider replacing “obstacles” with challenges, difficulties, or barriers encountered.

Usability: Do you mean compatibility issues and unstable internet connections? If so, change in text.

It would be helpful to provide contextual examples of clients where one needs to rely on family for physical assessments. It could be that, for the client profile in question, the preferred method recommended is face-to-face—a point to comment on in the Discussion section.

Table 3: It would be helpful to include mapping of the answers to the relevant survey questions, so the reader can link the two and has a point of reference.

With reference to sensory function (comment e below the table), I am finding it difficult to understand how one assesses sensory function using telerehabilitation methods accurately? Surely there must be validity and reliability issues with this method, please comment in the Discussion section.

Similarly, further down it refers to “clinician rated physical assessments.” Was there any concern for patient-reported outcomes? Especially patients who may have cognitive impairments or want to say what they think the professional wants to hear. Authors could comment on this point in the discussion.

##### Discussion

There is a reasonable discussion in light of the findings. Further to the comments marked for the Discussion previously, the authors could also discuss/elaborate on the following.

Paragraph 5: Comment on the potential implications of avoidance in some cases as in when carrying out assessments via video or telephone.

Next, the authors make a very valid point about “Understanding the actual versus perceived safety risks” but do not elaborate. I think that this is worth further elaboration.

Paragraph 6: The first line refers to “Technical and practical support from family members and carers...” What happened in cases where family/carer support was unavailable? How did professionals get around this challenge and any implications for the practice as a result?

Paragraph 7: Line 6 refers to training. Can the authors specify the kind of training required and which areas?

Last paragraph: The authors write “future surveys and qualitative studies should explore how experiences, attitudes and training needs evolve during and after the COVID pandemic.” What about the duration/competence of the clinical experience of the professional? Did this impact confidence? Is this a question that should be included in future surveys? Please comment.
